# Elevated BCAA Suppresses the Development and Metastasis of Breast Cancer

**DOI:** 10.3389/fonc.2022.887257

**Published:** 2022-06-16

**Authors:** Rui Chi, Chengcheng Yao, Si Chen, Yunxia Liu, Yanqi He, Jin Zhang, Lesley G. Ellies, Xuefeng Wu, Qian Zhao, Cixiang Zhou, Ying Wang, Haipeng Sun

**Affiliations:** ^1^ Department of Pathophysiology, Key Laboratory of Cell Differentiation and Apoptosis of Chinese Ministry of Education, Shanghai Jiao Tong University School of Medicine, Shanghai, China; ^2^ Shanghai Institute of Immunology, Department of Immunology and Microbiology, Shanghai Jiao Tong University School of Medicine, Shanghai, China; ^3^ Hongqiao International Institute of Medicine, Tongren Hospital, Shanghai Jiao Tong University School of Medicine, Shanghai, China; ^4^ Department of Pathology, School of Medicine, University of California, San Diego, La Jolla, CA, United States; ^5^ NHC Key Laboratory of Hormones and Development, Center for Cardiovascular Diseases, The Province and Ministry Co-Sponsored Collaborative Innovation Center for Medical Epigenetics, Chu Hsien-I Memorial Hospital & Tianjin Institute of Endocrinology, Tianjin Medical University, Tianjin, China

**Keywords:** branched-chain amino acid, breast cancer, NK cell, metastasis, N-Cadherin

## Abstract

Branched-chain amino acids (BCAAs) are the three essential amino acids including leucine, isoleucine, and valine. BCAA metabolism has been linked with the development of a variety of tumors. However, the impact of dietary BCAA intake on breast tumor progression and metastasis remains to be fully explored. Here, we unexpectedly find that the elevated BCAA, either in the genetic model or *via* increasing dietary intake in mice, suppresses the tumor growth and lung metastasis of breast cancer. The survival analysis shows that BCAA catabolic gene expression is strongly associated with long-term oncological outcomes in patients with breast cancer. In *Pp2cm* knockout mice in which BCAAs accumulate due to the genetic defect of BCAA catabolism, the breast tumor growth is suppressed. Interestingly, while the cell proliferation and tumor vasculature remain unaffected, more cell death occurs in the tumor in *Pp2cm* knockout mice, accompanied with increased natural killer (NK) cells. Importantly, increasing BCAA dietary intake suppresses breast tumor growth in mice. On the other hand, there are fewer lung metastases from primary breast tumor in *Pp2cm* knockout mice and the high BCAA diet-fed mice, suggesting high BCAA also suppresses the lung metastasis of breast cancer. Furthermore, low BCAA diet promotes lung colonization of breast cancer cells in tail vein model. The migration and invasion abilities of breast cancer cells are impaired by high concentration of BCAA in culture medium. The suppressed tumor metastasis and cell migration/invasion abilities by elevated BCAA are accompanied with reduced N-cadherin expression. Together, these data show high BCAA suppresses both tumor growth and metastasis of breast cancer, demonstrating the potential benefits of increasing BCAA dietary intake in the treatment of breast cancer.

## Introduction

Breast cancer is the most common malignant tumor among women worldwide (1). Current therapies for breast cancer include surgery, chemotherapy, radiotherapy, endocrine and target therapy. With improvements in the accuracy of diagnosis and the development of novel therapeutic agents, mortality from breast cancer has decreased. However, there are still no effective treatment strategies other than surgery for certain subsets of breast cancer, particularity the triple-negative breast cancer (TNBC) (2).

The reprogramming of metabolism represents an essential hallmark of cancers (3–6). Alterations of the uptake and metabolism of nutrients including glucose, amino acids, lipid, and so on, fulfill the tumor growth demands. In addition to glucose and lipid, the metabolic reprogramming of amino acid also plays critical role in tumor development. Glutamine, arginine, and glycine are the amino acids extensively studied in breast cancer and other types of cancer (7, 8). In TNBC, overexpressed SLC1A5, SLC7A5, and SLC6A14 promote glutamine metabolism and tumor growth (9). Promoting BCAA catabolism in TNBC suppresses protein translation, impairs mitochondrial function, and potentiates doxorubicin cytotoxicity (10). In addition to tumor growth, metabolic plasticity is also one important characteristic that distinguishes the tumor cells with high metastatic potentiality from non-metastatic tumor cells. Metastatic cancer cells usually operate multiple metabolic pathways concurrently to meet their adaptive requirements (11).

The role of the immune system in cancer pathogenesis has been a subject of great interest and the metabolic reprogramming also plays a key role in the immune cells (12). For example, lymphocytes require glucose to survive and the increased glucose consumption following activation supports their energetic and biosynthetic demands (13). The availability of specific nutrient affects the innate and/or adaptive immunity through manipulating T cell expansion and efficacy (14). Activated NK cells increase the expression of glycolytic enzymes and glucose transporters to promote glucose uptake and glycolysis (15). Therefore, it is unsurprising that the metabolic crosstalk between immune and cancer cells plays a key role in cancer development. The tumor-derived metabolites, such as adenosine and lactate, can limit the antitumor responses of immune cells (16–18). Tumor-driven glucose restriction in the tumor microenvironment (TME) reduces glucose availability and thus glycolysis in immune cells and impair their anti-tumor functions (19, 20). Similarly, tumor cells show increased amino acid consumption (21). Immune cells also show increased amino acid uptake and synergize with tumor-associated cells to create an amino acid-depleted microenvironment.

Branched-chain amino acids (BCAAs), leucine, isoleucine, and valine, are three essential amino acids. In mammals, BCAA homeostasis is controlled by their catabolic pathway. The first two steps of BCAA catabolism are shared by the three amino acids. BCAAs are initially transaminated by branched chain amino transferases (BCATs) to form branched chain α-ketoacids (BCKAs). BCAT1 encodes a cytoplasmic protein and is primarily expressed in the brain, whereas BCAT2 encodes a mitochondrial protein and is ubiquitously expressed. Irreversible initiation of BCKA oxidation occurs in the BCAA dehydrogenase (BCKDH) complex. The BCKDH complex is tightly regulated by phosphorylation/dephosphorylation. BCKDH kinase (BCKDK) phosphorylates BCKDHA to suppress BCKDH activity. The complementary activating dephosphorylation is carried out by the phosphatase PP2Cm encoded by the *PPM1K* gene (22).

Emerging evidence has linked BCAA metabolism closely with the growth and progression of various tumors, including glioblastoma, hepatocellular carcinoma (HCC), pancreatic ductal adenocarcinoma (PDAC), non-small cell lung cancer (NSCLC), acute myeloid leukemia (AML), and breast cancer (23–27). Recent studies have shown that BCKDK, the key regulator of BCAA catabolism, may act as a pro-metastatic factor in human colorectal cancer (28) and HCC (29). On the other hand, dietary BCAA supplementation suppresses liver tumor growth but promotes PDAC (30, 31). Other studies report that dietary BCAA levels are positively correlated with the development of tumors such as liver cancer (32) and PDAC (31) in mouse. It remains unclear whether BCAA intake influences breast tumor development and metastasis.

Numerus mechanisms have been implicated for BCAA’s function in cancer. BCAAs are building blocks for proteins and also stimulates mTOR signaling pathway to promote tumor growth (33). One recent study suggests that the highly-expressed BCAT1 in blast crisis chronic myeloid leukemia (BC-CML) re-amidate BCKA into BCAA, thus promoting the mTOR signaling pathway and the progress of BC-CML (34). Another report shows the degradation metabolism of BCAA is inhibited in a variety of tumor types, promoting the accumulation of intracellular BCAA, the activation of mTOR signaling pathway, and the occurrence and development of tumors (32). Increased BCAA degradation by BCAT1 overexpression is required for the proliferation, survival, and stemness maintenance of leukemia stem cells *via* the restriction on α-KG levels (27). Some cancer cells favor BCAA degradation as it provides precursors such as glutamate for the biosynthesis of fundamental building blocks to sustain cancer cell proliferation (24, 25).

Here we show that high BCAA suppresses the tumor growth and lung metastasis of breast cancer. The former is linked with enhanced immune function of NK cells and the latter is linked with the reduced expression of N-cadherin. Together, these data suggest the treatment of breast cancer may benefit from increasing BCAA dietary intake.

## Materials and Methods

### Western blot

SDS lysis buffer was used to harvest cells. For western blot, protein samples were boiled and separated on 8-12% SDS-PAGE gels. After blocking with 2% nonfat milk in Tris-buffered saline containing 1% Tween-20 for 1 hour, the membranes were incubated with specific primary antibodies overnight at 4°C. Antibody-bound proteins were detected by chemiluminescence (BIORAD, USA). The β-actin-HRP (Abways Technology, #AB2001), E-Cadherin (CST, #3195), N-Cadherin (CST, #13116), Vimentin (CST, #5741), PCNA (ABmart, #P30108s), Slug (CST, #9585), Cleaved caspase-3 (CST, #9661), Caspase-3 (CST, #9662), Anti-rabbit IgG-HRP (CST, #70742), Anti-mouse IgG-HRP (CST, #7076).

### Cell Lines and Culture Conditions

The human breast cancer cell lines LM2 and HEK293T cells were grown in Dulbecco’s modified Eagles’ medium (Hyclone, Beijing) supplemented with 10% fetal bovine serum (FBS, Gibco BRL, Gaithersburg, MD). The luciferase-expressing mouse mammary tumor cells 4T1 were cultured in RPMI-1640 Medium supplemented with 10% FBS. The mouse mammary tumor cells Py8119 were cultured in DMEM/F12(1:1) Medium containing Hydrocortisone (0.5μg/ml), mEGF (20ng/ml), Insulin(10μg/ml) and 10% FBS. All cells applied in this study were cultured at 37°C in a humidified 5% CO2 atmosphere.

### Cell Number and Viability Analysis by Trypan Blue

2 × 10^5^ Py8119 cells were plated in each well of 12-well plates. On the next day, the culture medium was changed to medium with different concentrations BCAA, and the cells were cultured for 48 hours. Cells were collected and 10μL cell suspension were taken, where 10μL of a 0.4% (m/v) previously prepared trypan blue solution was added. After stirring, the mixture was deposited on the cell counting plate. The cell number and viability were measured with automatic cell counter (Countstar). Independent triplicates were performed by analyzing three wells per group in each experiment.

### Orthotopic Allograft Mouse Model

Mouse mammary tumor cells Py8119 (1×10^5^) were suspended with Matrigel (3:1) in a total volume of 75μl and were orthotopically transplanted directly into the inguinal mammary fat pads of wild type or *Pp2cm* knockout female mice at the age of 6-7 weeks. The *Pp2cm* knockout mice were constructed by our laboratory (35). 3-4 weeks after injection, mice were euthanized and tumors were dissected and examined for tumor size and immunohistochemical staining. Tumor volume was calculated using the following formula: tumor volume (cubic millimeters (mm^3^)) = 0.5× (length × width^2^).

For the lung metastasis assay, the collected lungs were fixed with formalin and submitted for hematoxylin-eosin staining (H&E). The fixed lung tissue was sent to Google, a biological company, for subsequent paraffin embedding, sectioning, and staining experiments.

### TNBC Xenograft Mouse Model

Human breast cancer cells LM2 (1×10^5^) were suspended with Matrigel (3:1) in a total volume of 75μl and orthotopically transplanted into the inguinal mammary fat pads of NOD/SCID female mice at the age of 6 weeks. After injection, mice were feed with normal BCAA diet or high BCAA diet for 8 weeks. The high BCAA diet (Research Diets, Inc, A12030801) was 1.5 times higher than the normal BCAA diet (Research Diets, Inc, A11072001) (36). Eight weeks after injection, mice were euthanized and tumors were dissected and examined for tumor size and immunohistochemical staining. Tumor volume was measured weekly and calculated as before described.

### Tail Vein Injection Assay

The 5-6-week-old BALB/C mice were obtained from SLAC Laboratory Animals Company Limited, Shanghai, China. The purchased mice were transferred to Animal facility for 3 days and pretreated with a low BCAA diet (LBCAA, Research Diets, Inc, A12030802) or a normal BCAA diet (NBCAA, Research Diets, Inc, A11072001) for 2 weeks (36). BALB/C mice (n = 10, 11 per group) were injected in their tail veins with 5×10^5^ Luciferase-expressing 4T1 cells. After injection, BCAA diets were continued to the end of experiment. To investigate tumor metastasis, 12 days after injection, each mouse was intraperitoneally injected 200 mg/kg D-Luciferin. Bioluminescence was analyzed by IVIS system.

### Flow Cytometry

Tumor Dissociation Kit (Miltenyi Biotec, #130-096-730) was used for single cell suspensions from mouse tumor tissue. The single cell suspension of spleen was obtained by grinding, and white blood cells were obtained by 5% Ficoll density gradient centrifugation. Red Blood Cell Lysis Solution (10×) (Miltenyi Biotec, #130-094-183) was used to remove the erythrocytes. For cell-surface analysis, cells were stained with Fixable Viability Stain 780 (BD Horizon, #565388), anti-mCD45(BD Pharmingen, #561087), anti-mCD3e (BD Pharmingen, #557984), anti-mCD4 (BD Pharmingen, #561831), anti-mCD8a (BD Pharmingen, #561109), anti-CD11b (BD Pharmingen, #561098), anti-mF4/80 (BD Pharmingen, #743280), anti-mCD86 (BD Pharmingen, #564198) in recommended antibody concentrations and incubated at 4°C for 30 min. For the NK intracellular IFN-γ (BD Pharmingen, #562333) and granzymeB (Miltenyi Biotec, #130-101-356) cytokine staining, cells were fixed and permeabilized after stimulation with Leuko Act Cktl with GolgiPlug (BD Pharmingen, #550583) for 6 h. For the CD206 (BD Pharmingen, #565250) and NK1.1 (BD Pharmingen, #563220) staining, cells were also fixed and permeabilized. Cytofix/Cytoperm Soln Kit (BD Pharmingen, #554714) was used to fix and permeabilize the cells. Flow cytometry data was analyzed using FlowJo version 10.

### Wound Healing Cell Migration Assay

2×10^5^ LM2 cells were cultured in a six-well plate (5×10^5^ cells were cultured in a 6cm plate) until 90-100% confluence and then carefully scratched with a 10μl/200μl pipette tip. After washing three times with 1×PBS to remove detached cells, the culture medium was changed to BCAA gradient medium. The BCAA gradient medium was formulated with BCAA-free DMEM and BCAA chemicals. Images in 3 different wound fields were captured at respective time points (0h and 48h) to evaluate the migration of cells.

### Transwell Assay (Cell Migration and Invasion Assay)

Chambers (#3422, 8µm pore, Corning, NY, USA) without or with matrigel (#356234, BD Biosciences, CA, USA) were used to investigate migration and invasion ability of cells, respectively. Systems without Matrigel were used to measure the migration ability of cells. BCAA concentration gradient medium was used in the experiment. 2×10^4^ cells suspended in 100μL serum-free medium were seeded onto the upper chamber of 24-well plates, and 700μL of medium with 10% FBS was added to the lower chamber. 22 hours later, the medium was removed from the upper chamber. The cells on the membrane were fixed with 4% paraformaldehyde for 30 minutes, and the cells were stained with 0.1% of crystal violet (Sangon Biotech) overnight at 37°C. Then non-invading cells on the upper side of the chamber were gently removed thoroughly with a clean wet cotton swab and rinsed in clean water and dried overnight in oven at 37°C. Finally, the stained cells were counted by microscopy. Results represent the average number of cells in three fields per membrane and the experiment was repeated three times independently. As for cell invasion assays, the matrigel was diluted according to the manufacturer’s recommendations and added onto the chambers before seeding cells, then performed in the same manner as cell migration assays.

### Animals

All animal experiments were performed in accordance with relevant institutional and national guidelines and regulations of Shanghai Medical Experimental Animal Care Commission.

### Kaplan-Meier Plotter and Oncomine Analysis

The prognostic significance of the mRNA expression of BCAA catabolism genes in TNBC was evaluated using the Kaplan-Meier plotter (www.kmplot.com), an online database including gene expression data and clinical data. With the purpose to assess prognostic value of a specific gene, the patient samples were divided into two cohorts according to the median expression of the gene (high vs. low expression). The relapse-free survival (RFS) of TNBC patients was analyzed by using a Kaplan-Meier survival plot. Log rank p-value and hazard ratio (HR) with 95% confidence intervals were calculated. The Affymetrix ID of each gene in this study: *BCAT2* (ID203576 at), *BCKDK* (ID202030 at), *PPM1K* (ID244011 at).

The individual gene expression level of *BCAT2*, *BCKDK* and *PPM1K* was analyzed by Oncomine. The mRNA levels of cancer vs. normal patient datasets were compared. 1.25fold change, p-value=0.05, and top 10% gene rank were selected as threshold.

### Statistical Analysis

Statistical analysis was performed using GraphPad Prism 7.0 Software (GraphPad, San Diego, CA). The results were expressed as mean ± SD. 1-way ANOVA or a two-tailed Student’s t test was performed to analyze the statistically significance. P-values < 0.05 was considered as significant. *p < 0.05; **p < 0 01; ***p < 0.001.

## Results

### BCAA Metabolic Remodeling Is Strongly Associated With Breast Cancer Prognosis

**Figure 1:**
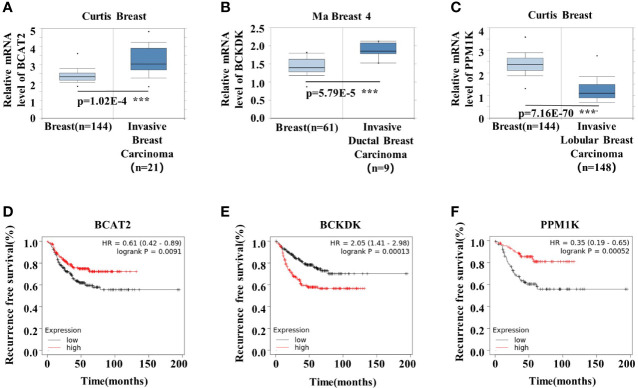
BCAA metabolic remodeling is strongly associated with breast cancer prognosis. **(A-C)** mRNA expression levels of BCAA catabolic enzymes were analyzed by Oncomine database: **(A)** *BCAT2*, **(B)** *BCKDK*, **(C)** *PPM1K*. **(D)** Kaplan-Meier RFS curves stratified by *BCAT*2 expression levels in 392 TNBC patients. The mean BCAT2 mRNA value was used to assign patients to two subgroups (ID203576 at). **(E)** Kaplan-Meier RFS curves stratified by *BCKDK* expression levels in 392 TNBC patients. The mean *BCKDK* mRNA value was used to assign patients to two subgroups (ID202030 at). **(F)** Kaplan-Meier RFS curves stratified by *PPM1K* expression levels in 176 TNBC patients. The mean *PPM1K* mRNA value was used to assign patients to two subgroups (ID244011 at). Mean ± SD, ***p < 0.001, Student’s t-test.

To understand the role of BCAA metabolism in breast cancer in human, we analyzed the BCAA metabolic gene expression in human breast tumors using Oncomine database. Among the genes involved in the initial 2 shared steps, the mRNA expression of *BCAT2* and *BCKDK* was significantly higher, whereas the expression of *PPM1K* was lower, in the invasive carcinoma than that in normal breast tissue (**Figures 1A–C**). The prognostic value of BCAA metabolic gene mRNA expression in breast cancer was assessed according to relapse-free survival (RFS) using Kaplan–Meier plotter. The Kaplan-Meier plotter revealed that the higher level of *BCAT2* was correlated with preferable RFS (**Figure 1D**). Increased expression of BCKDK was correlated with poor outcome in breast cancer patients (**Figure 1E**). Breast cancer patients with up-regulated *PPM1K* demonstrated better RFS (**Figure 1F**). These results suggest that BCAA metabolic reprogramming is strongly associated with outcome in breast cancer patients.

### Elevated BCAA Inhibits Breast Tumor Growth in the Orthotopic Allograft Model

**Figure 2:**
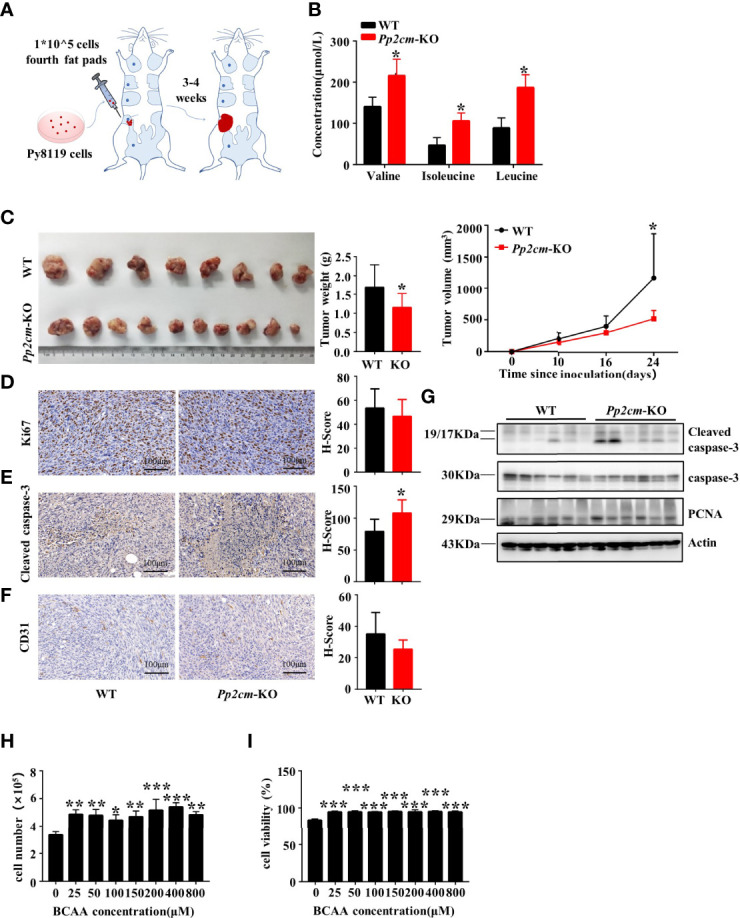
Elevated BCAA inhibits orthotopic tumor growth of breast cancer. **(A)** The orthotopic allograft model. **(B)** The serum BCAA concentration of tumor-bearing mice after 6 hours of fasting. Mean ± SD, *p < 0.05, Student’s t-test. **(C)** The tumor-bearing *Pp2cm*-WT/KO mice were euthanized at 21days and tumor were harvested (*Pp2cm*-WT, *n*=8; *Pp2cm*-KO, *n*=10). Tumor weight was measured at the end of the experiment and tumor volume was calculated using the following formula: tumor volume (cubic millimeters (mm^3^)) = 0.5× (length×width^2^). Mean ± SD, *p < 0.05, Student’s t-test. **(D)** Proliferation marker Ki67, **(E)** apoptosis marker Cleaved caspase-3, and **(F)** angiogenesis marker CD31 expression was analyzed by immunohistochemistry. Mean ± SD, *p < 0.05, Student’s t-test. **(G)** Western blot was used to analyze the protein expression of Cleaved caspase-3, caspase-3 and PCNA (n=6 in each group). **(H-I)** Py8119 cell number and viability assay using Trypan blue exclusion test after treatment with different concentrations of BCAA for 48 hours. Cell viability was calculated as the number of viable cells divided by the total number of cells. The BCAA gradient medium was prepared with 8000μM BCAA storage solution and custom BCAA-free DMEM. The storage solution was prepared from BCAA powder. BCAA treated groups (25μM, 50μM, 100μM, 200μM, 400μM, 800μM) were compared with the 0μM BCAA group. Mean ± SD, **p < 0.01, ***p < 0.001, ANOVA.

We investigated the impacts of elevated BCAA on breast tumor progression in *Pp2cm*-dificient mice in which BCAA accumulated due to the impaired BCAA catabolism (**Figure 2A**) (35). As expected, the plasma levels of BCAA were elevated in *Pp2cm*-dificient mice, compared with those in the control wildtype mice (**Figure 2B**). We constructed an orthotopic allograft model using Py8119, a kind of murine TNBC cell line (37), in *Pp2cm*-dificient and control mice. Unexpectedly, the tumor growth was significantly suppressed in *Pp2cm* knockout mice (**Figure 2C**).

To explore the underling mechanism, we assessed the cell proliferation, cell death, and angiogenesis in tumor tissues in *Pp2cm*-deficient and control mice. Immunohistochemistry staining showed no significant differences in the expression of proliferation markers Ki67 (**Figure 2D**) and proliferating cell nuclear antigen (PCNA) (**Figure 2G**) and angiogenesis marker CD31 (**Figure 2F**). Unexpectedly, the apoptosis marker cleaved caspase-3 was significantly up-regulated in the tumor in *Pp2cm*-deficient mice, compared with those in the control wildtype mice (**Figures 2E, G**).

We assessed the direct impacts of high level BCAA on breast cancer cell growth and death *in vitro*. The concentration of BCAA in Dulbecco’s Modified Eagle Medium (DMEM) is 800 μM, higher than their fasting plasma levels (~50-200 μM) under physiological conditions. Py8119 cells were cultured with different concentrations of BCAA in medium. High level of BCAA did not suppress breast cancer cell growth (**Figure 2H**) or induce cell death (**Figure 2I**). Therefore, the increased cell death in tumors in *Pp2cm*-deficient mice was unlikely resulted from the direct effects of BCAA on cancer cells. Together, these results suggested high BCAA suppressed TNBC tumor growth by promoting tumor cell death *via* an indirect mechanism.

### Elevated BCAA Enhances the Activity of NK Cells

**Figure 3:**
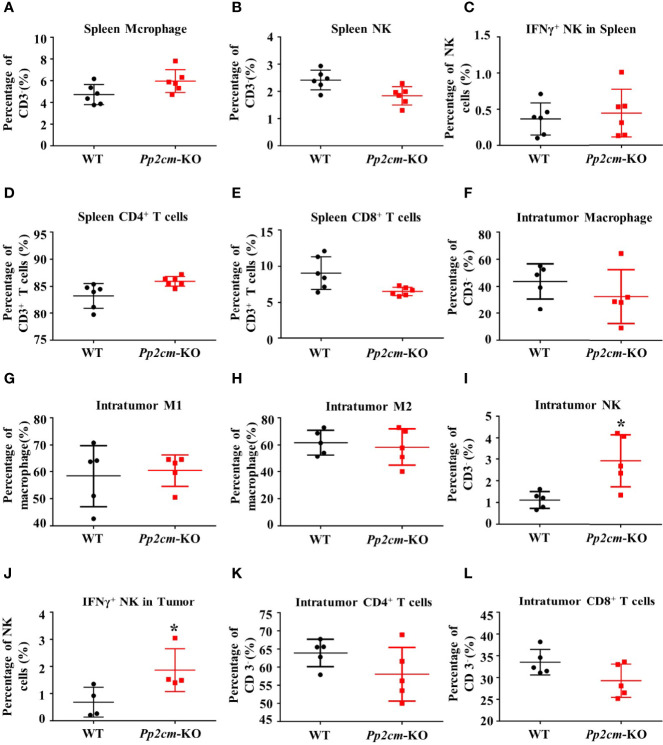
High BCAA enhances NK cell immune function in tumor-bearing mice. **(A–E)** Flow cytometry was used to analyze the percentage of immune cells including macrophage, NK cell, IFN-γ^+^ NK cell, CD4^+^ T cell, and CD8^+^ T cell in spleen. **(F–L)** Flow cytometry was used to analyze the percentage of intratumor macrophage, type-I macrophage, type-II macrophage, CD4^+^ T cell, CD8^+^ T cell, NK cell, and IFN-γ^+^ NK cell. Mean ± SD, *p < 0.05. Student’s t-test.

Immune system plays an important anti-tumor role in the development of cancer. We then analyzed the immune responses to TNBC tumor in *Pp2cm*-deficient mice. The populations of macrophage, NK cells, CD4^+^ T cells, CD8^+^ T cells, as well as the population of NK cells expressing interferon γ (IFN-γ) showed no significant changes in the spleens of *Pp2cm*-deficient mice, compared with those in control mice (**Figures 3A–E**). Among the tumor-infiltrating immune cells, no difference was detected for the populations of macrophage, type-I macrophage, type-II macrophage, CD4^+^ T cells, and CD8^+^ T cells (**Figures 3F–L**). However, the populations of NK cells and NK cells expressing IFN-γ were significantly increased in the tumors in *Pp2cm* knockout mice (**Figures 3I, J**). These results suggested that high BCAA enhanced the activity of tumor-infiltrating NK cells which could kill cancer cells and inhibit tumor development.

### Increasing Dietary BCAA Intake Suppresses Breast Tumor Growth in the Orthotopic Xenograft Model

**Figure 4:**
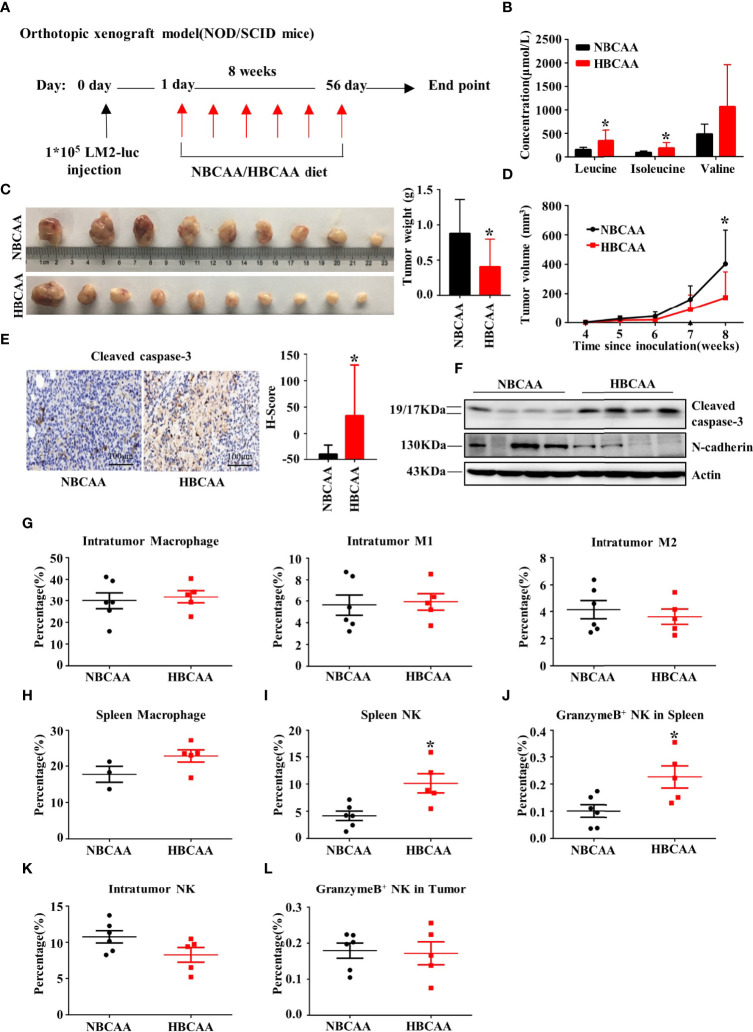
Increasing dietary BCAA intake suppresses tumor growth in the orthotopic xenograft model. **(A)** Orthotopic xenograft model (normal BCAA Diet, *n*=9; high BCAA Diet, *n*=10). **(B)** The serum BCAA concentration of tumor-bearing mice. **(C)** Tumors were harvested and tumor weight was measured at the end of the experiment. **(D)** Tumor volume was measured per week and calculated using the following formula: tumor volume (cubic millimeters (mm^3^)) = 0.5× (length × width^2^). **(E)** Apoptosis marker Cleaved caspase-3 were analyzed by immunohistochemistry (*n*=9 in each group). **(F)** Western blot to detect Cleaved caspase-3 and N-cadherin expression of tumor tissues. **(G)** The percentage of macrophage, type-I macrophage and type-II macrophage in tumor was analyzed by flow cytometry. **(H)** The percentage of macrophage in spleen. **(I)** The percentage of NK cell in spleen. **(J)** The percentage of NK cell expressing Granzyme B in spleen. **(K)** The percentage of NK cell in tumor. **(L)** The percentage of granzyme B^+^ NK cells in tumor. Mean ± SD, *p < 0.05, Student’s t-test.

We next investigated whether increasing dietary BCAA intake could affect breast cancer progression in an orthotopic xenograft model. The human breast cancer cell LM2 was injected into the inguinal mammary fat pad of female non-obese diabetic/severe combined immunodeficiency (NOD/SCID) mice. Mice were fed with different BCAA diets for 8 weeks (**Figure 4A**). The plasma concentrations of BCAA in the high-BCAA diet (HBCAA)-fed mice were significantly higher than those in the normal-BCAA diet (NBCAA)-fed group (**Figure 4B**). Importantly, the tumor mass in the HBCAA-fed mice was significantly smaller than that in the NBCAA-fed group (**Figure 4C**). HBCAA significantly inhibited the rate of tumor growth (**Figure 4D**). In agreement with the orthotopic allograft model (**Figures 2E, G**), significantly up-regulated apoptosis marker in the orthotopic xenograft tissues of HBCAA group was observed, compared with that of NBCAA group (**Figures 4E, F**).

In NOD/SCID mice, the adaptive immune system is deficient, accompanied with normal macrophage and low NK cell activity. Macrophage population showed no difference in tumor (**Figure 4G**) or spleen (**Figure 4H**) between HBCAA and NBCAA groups. However, HBCAA feeding increased the population of NK cells (**Figure 4I**) and NK cells expressing granzyme B (**Figure 4J**) in spleen. The populations of tumor-infiltrating NK cells (**Figure 4K**) and NK cells expressing granzyme B (**Figure 4L**) were not significantly changed.

### High BCAA Inhibits the Lung Metastasis of Breast Cancer

**Figure 5:**
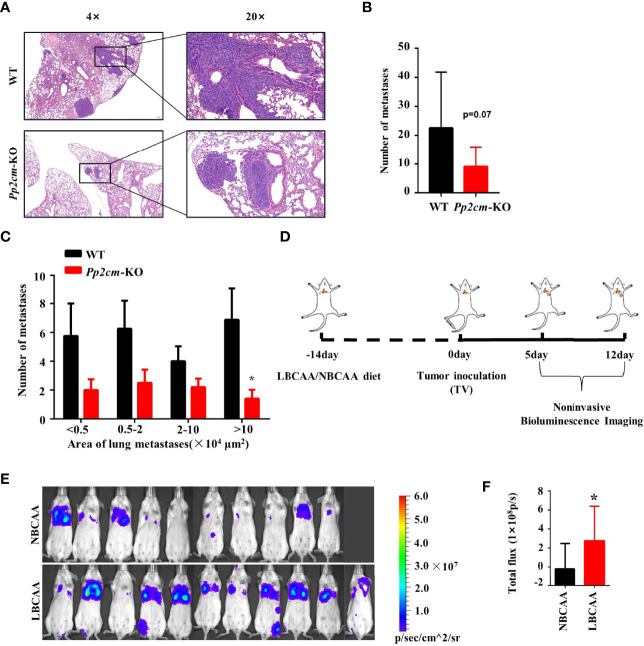
High BCAA inhibits the lung metastasis of breast cancer. **(A)** Representative images of H&E staining of lung tissues from tumor-bearing *Pp2cm*-WT/KO mice. **(B)** Statistical analysis of the total number of lung metastases (WT, *n*=8; *Pp2cm*-KO, *n*=10). **(C)** Statistical analysis by size of metastases. **(D)** A lung metastasis mouse model of breast cancer by the tail vein injection in mice fed low BCAA diet (LBCAA) or normal BCAA diet (NBCAA) **(E)** Detection of lung metastases at 13th day by Bioluminescence Imaging in mice from D (NBCAA, *n*=10; LBCAA, *n*=11). **(F)** Statistics of caudal vein lung metastasis. Mean ± SD, *p < 0.05, Student’s t-test.

The vast majority of breast cancer deaths are due to metastasis but not the primary tumor (38). To gain insight into the impacts of elevated BCAA on breast cancer metastasis, the tumor metastases in lung tissues was analyzed in the *Pp2cm* knockout and wildtype mice bearing tumor under inguinal mammary fat pads. Interestingly, the lung metastases in *Pp2cm* knockout mice were less than those in the control group (**Figures 5A–C**).

In addition to the genetic mouse model in which BCAA was elevated, we also analyzed the impacts of altering BCAA dietary intake on breast cancer metastasis. The tail-vein-metastasis model was established by injecting luciferase-expressing 4T1 cells in wildtype mice, and low BCAA diet significantly promoted lung colonization of breast cancer cells in this model (**Figures 5D–F**). Overall, these results provide evidence that high BCAA inhibits lung metastasis of breast cancer.

### High BCAA Inhibits Breast Cancer Cell Migration, Invasion, and the Expression of N-Cadherin

**Figure 6:**
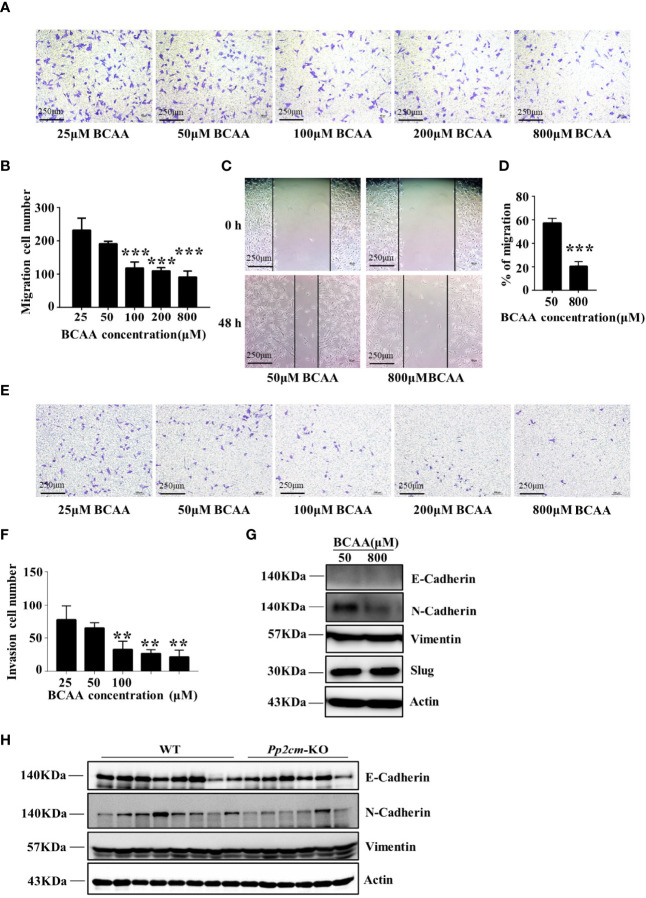
High BCAA inhibits the ability of breast cancer cell migration, invasion, and the expression of N-Cadherin. **(A)** Migration ability of LM2 cells treated with different concentrations of BCAA analyzed by transwell assay (22 hours). **(B)** Statistics of the number of migration cells in transwell assay. All BCAA treated groups (50μM, 100μM, 200μM, 800μM) were compared with 25μM BCAA group. ***p < 0.001, one-way ANOVA. **(C)** Wound healing assay was used to detect the migration ability of LM2 cells treated by different concentrations of BCAA (48 hours). **(D)** Statistics of the mobility ratio in wound healing assay. **(E)** Invasion ability of LM2 cells treated by different concentrations of BCAA was detected by transwell assay (22 hours). **(F)** Statistics of the number of invasion cells in transwell assay. All BCAA treated groups (50μM, 100μM, 200μM, 800μM) were compared with 25μM BCAA group. **p < 0.01, ANOVA. **(G)** N-Cadherin, E-Cadherin, Vimentin, and Slug expression was analyzed by Western blot in LM2 cells. **(H)** E-Cadherin, N-Cadherin, and Vimentin expression in tumor tissues was detected by Western blot.

To determine whether BCAA directly affected breast cancer cell migration, the transwell assay was conducted with different concentrations of BCAA in culture medium. The results showed that the migration of LM2 was inhibited when BCAA level increased (**Figures 6A, B**). Wound healing assay also showed high BCAA in medium inhibited the migration ability of breast tumor cells (**Figures 6C, D**). In addition, the invasion ability of LM2 was also suppressed by high level of BCAA (**Figures 6E, F**). PP2cm expression is low in LM2 cell. It remains unclear whether inactivation of PP2Cm makes the same impacts on breast cancer cell migration and invasion.

The epithelial-to-mesenchymal transition (EMT) is a biological process strongly associated with tumor progression, invasion, and metastasis (39, 40). As the concentration of BCAA increased in the culture medium, the expression of N-Cadherin, a key mesenchymal marker, dramatically decreased in LM2 cells (**Figure 6G**). The expression of N-Cadherin was also decreased in tumor tissues of HBCAA-fed mice in the orthotopic xenograft model (**Figure 4F**). On the other hand, the expression of E-Cadherin, Slug, and Vimentin showed no difference between different BCAA groups (**Figure 6G**). Furthermore, N-Cadherin expression was down-regulated in the breast tumor tissues in *Pp2cm* knockout mice compared with that in control mice, but the expression of E-Cadherin and Vimentin was not changed (**Figure 6H**).

## Discussion

The current study shows BCAA metabolic reprogramming is strongly associated with TNBC patient survival. In addition, elevated BCAA unexpectedly suppresses the growth and metastasis of breast tumor. The suppressed tumor growth is accompanied with enhanced NK cell activity. The reduced metastasis is accompanied with lower cancer cell migration and N-cadherin expression.

The functions of tumor infiltrating immune cells rely on nutrients in TME. However, nutrients are often limited in TME due to the delivery barriers and the competition among cancer cells, immunocytes, and other cell types. Tumor cells often outcompete immunocytes for nutrients, leading to impeded immune function and tumor immunological evasion (41, 42). Therefore, it is tempting to speculate that the elevated BCAA enhances NK cell activity in breast tumors *via* overcoming the BCAA inefficiencies in TME.

The mechanism underlying BCAA-promoted NK activity remains to be investigated. mTOR signaling pathway can be activated by BCAA (33). mTORC1 activity is essential for NK cell development and activation-induced functional responses in mature NK cells, including the expression of effector molecules IFN-γ and granzyme B (15, 43, 44). In the current study, high BCAA increases the secretion of IFN-γ and granzyme B by NK cells in tumor-bearing mice. Thus, BCAA may enhance NK cell responses by regulating mTOR signaling pathway. In addition, elevated BCAA could provide more protein building blocks for NK cells.

The BCAA-suppressed breast tumor metastasis is accompanied with lower N-cadherin expression. N-cadherin is the key protein for tumor invasion. Studies have demonstrated that increased N-cadherin expression enhances the migratory and invasive capacities of multiple types of epithelial cancer cells *in vitro* (45). Moreover, the facilitation of tumor distant metastasis by N-cadherin expression has been demonstrated in mouse tumor models of breast cancer, pancreatic cancer, prostate cancer, and melanoma (46, 47). Therefore, N-cadherin might act as an important mediator for BCAA-suppressed cancer metastasis. Meanwhile, how BCAA suppresses N-cadherin expression remains unclear.

The survival analysis suggests that BCAA metabolic reprogramming is strongly associated with the outcome of breast cancer patients. The increased *BCKDK* and the downregulated *PPM1K* expression indicate suppressed BCAA catabolism is associated with poor survival. This correlation is in line with previous report showing suppressed BCAA catabolism promotes cancer cell proliferation (32). Based on this correlation, increasing BCAA intake would likely promote tumor progression. However, our data show that increasing BCAA intake suppresses tumor progression. This unexpected result has been correlated with enhanced immune function by elevated BCAA in animals. Thus, when BCAA is elevated, their pro-immune impacts on immunocytes overcomes their pro-tumor impacts on cancer cells, leading to suppressed tumor growth. In this way, even the impaired BCAA catabolism in tumor cells is correlated with poor survival, the elevated environmental BCAA could be correlated with better survival.

Given that commercial BCAA products are easily available, these data suggest increasing BCAA intake may provide a practical dietary approach to slow the tumor progression and boost the immunotherapy of breast cancers. Further studies of BCAA’s impacts on other types of cancers will provide more insights.

## Data Availability Statement

The raw data supporting the conclusions of this article will be made available by the authors, without undue reservation.

## Ethics Statement

The animal study was reviewed and approved by Shanghai Medical Experimental Animal Care Commission.

## Author Contributions

HS, YW, and CZ designed the project and directed the research. RC, CY, SC, YL, YH, and JZ performed the research. RC, SC, CY, YW, CZ, and HS analyzed the data. LE, XW, and QZ helped to design the overall study and analyzed the data. All authors contributed to the manuscript preparation. All authors contributed to the article and approved the submitted version.

## Funding

This work was supported by Ministry of Science and Technology of China (2019YFA0802503), the National Natural Science Foundation of China (92057107, 81570717, 31900819), The National Key Research and Development Program of China (2021YFC2701800, 2021YFC2701804), Collaborative Innovation Program of Shanghai Municipal Health Commission (2020CXJQ01), Shanghai Collaborative Innovation Center for Translational Medicine (TM202112), Science and Technology Innovation Program of Shanghai Municipal Government (19411950500), and the Science and Technology Commission of Shanghai Municipality (16JC1404400).

## Conflict of Interest

HS participated in an advisory board for Ramino Bio Ltd.

The remaining authors declare that the research was conducted in the absence of any commercial or financial relationships that could be construed as a potential conflict of interest.

## Publisher’s Note

All claims expressed in this article are solely those of the authors and do not necessarily represent those of their affiliated organizations, or those of the publisher, the editors and the reviewers. Any product that may be evaluated in this article, or claim that may be made by its manufacturer, is not guaranteed or endorsed by the publisher.
